# Gender-specific association between circulating 25-hydroxyvitamin D levels and diabetic retinopathy in patients with type 2 diabetes mellitus

**DOI:** 10.3389/fendo.2025.1541961

**Published:** 2025-04-29

**Authors:** Jing He, Jingrui Kang, Zhou Mei, Xiaohui Zhou, Yingchuan Yin, Cuiling Zhu

**Affiliations:** ^1^ Department of Endocrinology, The Third People’s Hospital of Hefei, Hefei Third Clinical College of Anhui Medical University, Hefei, China; ^2^ Department of Endocrinology, Cangzhou Hospital of Integrated Traditional Chinese Medicine and Western Medicine of Hebei, Hebei, China; ^3^ Department of Endocrinology and Metabolism, Shanghai Tenth People’s Hospital of Tongji University, School of Medicine, Shanghai, China

**Keywords:** type 2 diabetes, diabetic retinopathy, 25-hydroxyvitamin D, total testosterone, gender-specific difference

## Abstract

**Background:**

Diabetic retinopathy (DR), one of the most common microvascular complications of diabetes mellitus (DM), remains to be a major driver of vision loss worldwide. Vitamin D has been reported to be involved in DR pathogenesis, but results have been inconsistent. We aimed to explore the relationship between blood 25-hydroxyvitamin D, 25(OH)D, level and the risk of DR in patients with type 2 diabetes (T2DM).

**Methods:**

A total of 535 adults with T2DM from our department were included. Demographic information, biochemical data, 25(OH)D, and sex hormones were collected. Fundus photography was performed to determine the presence of DR. Participants were grouped into the DR group and the non-DR (NDR) group according to the fundus examinations and four groups based on serum 25(OH)D levels as follows: normal, insufficient, deficient, and severely deficient. Multivariate logistic regression analysis was used to evaluate the association between 25(OH)D and risk of DR.

**Results:**

Males but not females with DR had significantly decreased levels of 25(OH)D (16.4 ± 5.6 ng/ml vs. 21.0 ± 5.0 ng/ml, *P* = 0.001) and increased proportion of severe 25(OH)D deficiency (14.8% vs. 6.7%, *P* = 0.022) compared to those without DR. Likewise, there was a gradually increasing percentage of DR with the reduction of 25(OH)D levels only in males (35.7%, 44.4%, 53.2%, 70.3%, *P* = 0.022). Intriguingly, total testosterone (TT) levels decreased markedly in males with DR (12.9 ± 5.2 nmol/L vs. 14.2 ± 5.5 nmol/L, *P* = 0.035) compared to their counterparts and correlated positively with 25(OH)D (β = 0.161, *P* = 0.007), which did not occur in females. After multivariate adjustment, we observed a significant inverse association between serum 25(OH)D and the occurrence of DR in males, showing that the adjusted ORs (AORs) and 95% confidence interval for DR were 0.233 (0.070–0.779) in the normal group, 0.280 (0.103–0.756) in the insufficient group, and 0.477 (0.196–1.164) in the deficiency group with the severely deficient group as a reference (*P_-trend_* = 0.003). However, such a significant association was not observed in females (*P_-trend_* = 0.137).

**Conclusion:**

We concluded a gender-specific relationship between serum 25(OH)D and the incidence of DR in T2DM, supported by a significant inverse association between serum 25(OH)D and DR only in males, which could be mediated by a marked reduction in TT levels.

## Introduction

Diabetic retinopathy (DR) is one of the most common and severe microvascular complications of type 2 diabetes mellitus (T2DM) and a leading driver of blindness and vision impairment among the adult working population, especially in developed countries (1, 2). It is estimated that 103.12 million adults worldwide are suffering from DR in 2020, which is projected to increase to 160.50 million by 2045, with the world DR incidence increasing by 55.6% from 2020 to 2045 (1). Additionally, the estimated healthcare cost of DR in Indonesia was $2.4 billion in 2017 and $8.9 billion in 2025, approximately threefold increase in the cost, causing serious consequences for patients and an enormous economic strain on the healthcare system (3). The origins of DR are derived from microvascular impairment, vasodilation, and exudates, which gradually progress to a complicated stage and subsequently result in retinal detachment. It progresses through multiple pathophysiological pathways, including oxidative stress, chronic inflammation, vascular endothelial growth factor stimulation in the retinal blood vessels, and immune modulation (4). Current research has reported that there are many factors leading to DR, but its pathogenesis remains unclear. Therefore, identifying the main risk factors contributing to the development of DR in T2DM is essential for early detection, intervention, and management of DR, which will be more cost-effective for public health and healthcare costs.

The onset of DR is affected by age at diagnosis, a longer diabetes duration, poor glycemic control, hypertension, hyperlipidemia, obesity, and cardiovascular disease (2, 5, 6). Intriguingly, emerging evidence from human and animal studies has shown that vitamin D may be involved in DR pathogenesis (7, 8). Vitamin D, regarded as an essential nutrient crucial in bone metabolism, has been validated to exert various biological effects, including antioxidant defense, immune modulation, anti‐inflammatory action, and anti-angiogenesis (7–9), which plays a key role in the pathogenesis of DR (10). Indeed, the 25-hydroxyvitamin D, 25(OH)D, a stable vitamin D metabolite, is regarded as a reliable biomarker of vitamin D status and a widely acceptable measurement in clinical. A prospective study including 14,709 participants with T2DM showed a significant inverse association between serum 25(OH)D levels and risk of DR (11). Similarly, in one large meta-analysis including 15 observational studies involving 17,664 subjects with T2DM, patients with serum 25(OH)D deficiency had a significantly increased risk of DR [OR = 2.03, 95% confidence interval (CI): 1.07, 3.86], and patients with DR had a significantly reduced 25(OH)D level as opposed to their controls (12). Another meta-analysis including fourteen observational studies with 10,007 participants also showed a significant inverse association between DR and 25(OH)D in diabetic patients (OR = 1.27, 95% CI: 1.17, 1.37) (13). Recent experimental studies suggest that vitamin D protects against DR through its anti-inflammatory and anti-angiogenic properties. Proinflammatory cytokines such as interleukin-6 (IL-6) and tumor necrosis factor–alpha (TNF-α) are increased in patients with T2DM, and vitamin D addition has been shown to reduce the production of these proinflammatory cytokines in the retina by enhancing its anti-inflammatory properties (14). In an oxygen-induced ischemic retinopathy mouse model, calcitriol (the active metabolite of vitamin D) could protect against DR in a dose-dependent manner, acting as a potent inhibitor of retinal neovascularization (8). Furthermore, vitamin D supplementation among subjects with T2DM showed a significant reduction in TNF-α and high-sensitivity C-reactive protein (hs-CRP) (15), which can lead to vascular occlusion and synthesis of new vessels, accelerating the progression of DR (16). These findings revealed that vitamin D might protect against the development of DR through various pathogenesis. Notably, Jee et al. (17) found that 25(OH)D level correlated inversely with the prevalence of DR in men but not in women among a representative Korean population. Another inconsistent result from Reddy et al. (18) found that the proportion of 25 (OH)D deficiency was significantly higher in diabetic patients with or without DR (66% and 63%) compared to those without T2DM, whereas no significant differences were observed between the groups with diabetes. Some other studies did not find any association between 25(OH)D and the risk of DR in T2DM (19, 20). Collectively, the correlation between 25(OH)D and DR is complex, which needs to be further investigated and testified.

In view of significant gender differences in circulating 25(OH)D levels (21) and controversial results regarding the relationship between serum 25(OH)D and DR incidence (17, 19), the current study was performed to explore the possible association between 25(OH)D levels and DR among the population with T2DM stratified by gender, aiming to provide individualized treatment options for these patients.

## Materials and methods

### Study design and participants

**Figure 1:**
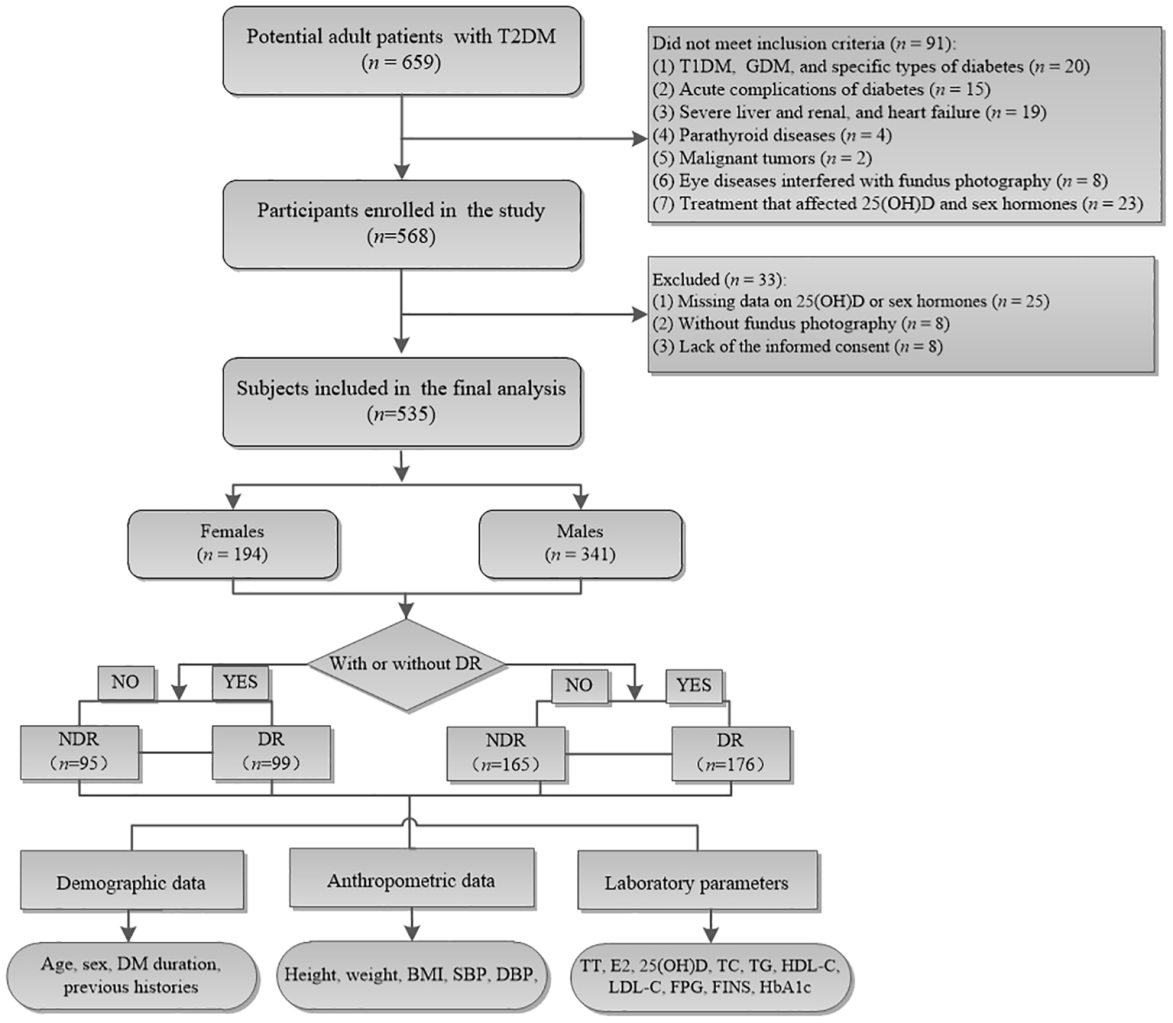
Flowchart of the study cohort. Among the 659 potential adult patients, 124 who did not meet the inclusion criteria were excluded and finally 535 were enrolled in the current analysis. NDR, patients without diabetic retinopathy; DR, patients with diabetic retinopathy.

This cross-sectional study was performed at the Department of Endocrinology, The Third People’s Hospital of Hefei, Hefei Third Clinical College of Anhui Medical University in China between January 2020 and November 2024. A total of 659 adult patients with T2DM who met the following inclusion criteria were consecutively enrolled: (1) aged 18–75 years old, (2) a confirmed diagnosis of T2DM, and (3) undergoing fundus photography. The exclusion criteria were as follows: (1) T1DM, GDM, and other specific types of diabetes; (2) acute complications of diabetes; (3) severe liver, renal, and heart failure; (4) parathyroid diseases; (5) malignant tumors; (6) cataract, glaucoma, and other eye diseases that affected the results of fundus photography; (7) previous or current treatment with drugs or nutrition supplements that might affect 25(OH)D and sex hormone levels, such as zoledronic acid, calcitonin, estrogen, and estrogen receptor modulator; (8) without a fundus photography; (9) missing data on 25(OH)D or sex hormone levels; and (10) lack of informed consent prior to this study. Finally, 535 participants (341 males and 194 females) were analyzed in this study (**Figure 1**). All participants provided written informed consent prior to this study. This study was approved by the Research Ethics Review Committee of The Third People’s Hospital of Hefei, Hefei Third Clinical College of Anhui Medical University in China.

### Anthropometric measurements

Demographic and clinical information of age, sex, height, body weight, systolic blood pressure (SBP), diastolic blood pressure (DBP), duration of diabetes mellitus (DM), and medication history were recorded by trained physicians. The body mass index (BMI) was calculated as body weight (kg) divided by the square of height (m^2^). BP was measured after at least a 10-min rest, with the patient in a seated position by experienced physicians. Three readings were taken, each at least 2 min apart, and then the mean value of the readings was calculated.

### Clinical biochemical measurements

Following an overnight fast, venous blood samples were drawn from all the participants on the same day as the fundus photograph. Subsequently, the levels of total cholesterol (TC), triglycerides (TG), low-density lipoprotein cholesterol (LDL-C), high-density lipoprotein cholesterol (HDL-C), fasting plasma glucose (FPG), fasting insulin (FINS), fasting C peptide (FCP), glycosylated hemoglobin (HbA1c), total testosterone (TT), and estradiol (E2) were measured. Circulating 25(OH)D concentration was measured using an electrochemiluminescence assay (Roche, Switzerland). Based on the grouping criteria for serum 25(OH)D levels defined in the research by Gverović et al. (22), the corresponding status of 25(OH)D concentrations was divided into the following four groups. Serum 25 (OH) D < 10 ng/ml was categorized as severely deficient, 10 ng/ml ≤ 25 (OH) D < 20 ng/ml as deficient, 20 ng/ml ≤ 25 (OH) D < 30 ng/ml as insufficient, and 25 (OH) D ≥ 30 ng/ml as normal. However, we acknowledge that regional variations exist in vitamin D levels due to differences in sunlight exposure, dietary habits, and supplementation practices. Homeostasis model assessment of IR (HOMA-IR) was computed as FPG (mmol/L) × FINS (mU/L)/22.5, described by Matthews et al. (23) All the laboratory measurements were carried out in our department using standard methodologies.

### Definition and subgroup assignment

The definition of diabetes was assigned to those with diagnosed diabetes by a physician, or use of insulin and hypoglycemic medication, or presence of fasting plasma glucose level ≥ 7.0 mmol/L, casual plasma glucose level ≥ 11.1 mmol/L, or 2‐h plasma glucose level in an oral glucose tolerance test (OGTT) ≥ 11.1 mmol/L, or HbA1c level ≥ 6.5% (24). The diagnosis of T2DM was made according to the guideline for the prevention and treatment of T2DM mellitus in China (2020 edition) (25). DR was detected by fundus photography according to characteristic clinical manifestations including microaneurysm, hemorrhage, hard exudates, soft exudates, intra-retinal microvascular anomalies (IRMA), neovascularization of the retina elsewhere (NVE), and neovascularization of the disc (NVD), and even retinal detachment (TRD), which was defined by the Early Treatment Diabetic Retinopathy Study (ETDRS) (26). All the fundus photograph images were performed and analyzed by two different experienced physicians and a trained ophthalmologist who were blinded to the clinical statuses of the subjects. As a result, individuals were stratified into the DR group (*n* = 275) and the NDR group (*n* = 260) based on the results of fundus photography.

### Statistical analysis

All statistical analyses were performed using SPSS 23.0 software (SPSS Inc., Chicago, IL, USA) or the GraphPad Prism 6.0 project. Data were described using means ± standard deviation (SD) or medians (interquartile ranges) for continuous variables and absolute and relative proportions (*n*, %) for categorical variables. An independent Student’s t-test for normally distributed variables or a non-parametric test for non-normally distributed data was performed to compare the differences between groups. Categorical variables were analyzed using the chi-squared test or Fisher’s exact test when appropriate. Non-normally distributed data were logarithmically transformed to normality when needed. Bivariate correlation analysis was used to explore the correlation of 25(OH)D levels with metabolic-related indices in both males and females. Multivariate linear regression analysis was performed, adjusting for potential confounders including age, sex, BMI, diabetes duration, HbA1c, SBP, DBP, TC, TG, LDL-C, FPG, FINS, FCP, and HOMA-IR in both genders. To evaluate the effect of serum 25(OH)D levels on the DR prevalence, binary logistic regression analyses were used to determine the correlation of 25(OH)D level with DR in three different models: (1) for age and BMI; (2) for age, BMI, FPG, and FINS; and (3) for age, BMI, FPG, FINS, HOMA-IR, TT, and DM duration. Unadjusted and adjusted odds ratios (ORs) and 95% CI were calculated. A two-tailed value of *P* < 0.05 was considered statistically significant. Regarding the missing data, we conducted a complete-case analysis, excluding subjects with missing key variables. The overall missing data rate was 5.6%. Sensitivity analysis was performed using multiple imputation methods, and the results remained consistent.

## Results

### Baseline characteristics of study participants

T2DM was diagnosed in 535 (81.1%) of the 659 eligible subjects and included in the current study analysis. The mean age of the included participants was 61.8 ± 11.4 years, and 63.7% (*n* = 341) of whom were males. Based on the typical clinical manifestation of DR, 275 (51.4%) of patients were diagnosed as DR. As depicted in **Table 1**, the baseline characteristics of the enrolled 535 patients stratified by DR status were presented. Subjects with DR were more likely to be elder (*P* < 0.001) and had significantly longer DM duration (*P* < 0.001), higher SBP (*P =* 0.020), TG (*P =* 0.011), FINS (*P =* 0.009), and HOMA-IR (*P =* 0.022), as well as decreased BMI (*P =* 0.021), FCP (*P =* 0.010), and 25(OH)D level (*P =* 0.001) than those without DR, while sex hormone levels including E2 and TT did not change significantly between the two groups. After stratifying by gender, significantly increased age, DM duration, SBP, FINS, HOMA-IR, and reduced FCP levels were still seen in patients with DR than their counterparts in both genders (all *P* < 0.05), whereas increased TG (*P =* 0.006) and reduced TT levels (*P* = 0.035) were only observed in males but not in females (**Table 2**). Likewise, we found that male diabetic patients with DR showed significantly reduced 25(OH)D level (16.4 ± 5.6 ng/ml vs. 21.0 ± 5.0 ng/ml, *P* = 0.001) as opposed to their controls, which was nonsignificant in females (all *P* > 0.05). However, there was no significant difference in BMI, DBP, TC, HDL-C, LDL-C, FPG, E2, and HbA1c between the two groups in either gender (all *P* > 0.05).

**Table 1:** Demographic and clinical characteristics of study cohort stratified by DR status.

Parameters	NDR (*n* = 260)	DR (*n* = 275)	*P*-value
Age (year)	59.7 ± 13.3	63.8 ± 8.8	< 0.001
Sex (M/F)	(165/95)	(176/99)	0.897
DM duration (year)	10.2 ± 6.7	14.3 ± 7.6	< 0.001
BMI (kg/m^2^)	25.5 ± 4.5	24.7 ± 3.4	0.021
SBP (mmHg)	134 ± 22	139 ± 19	0.020
DBP (mmHg)	75 ± 11	74 ± 11	0.217
TC (mmol/L)	4.3 ± 1.1	4.3 ± 1.0	0.813
TG (mmol/L)	1.7 ± 1.1	2.0 ± 1.3	0.011
HDL-C (mmol/L)	1.0 ± 0.3	1.1 ± 0.3	0.502
LDL-C (mmol/L)	2.5 ± 0.9	2.6 ± 0.9	0.555
FPG (mmol/L)	8.3 ± 3.4	8.1 ± 3.1	0.580
FINS (mU/L)	13.2 (10.1)	25.6 (11.5)	0.009
FCP (mmol/L)	2.2 ± 1.3	2.0 ± 1.5	0.010
HbA1c (%)	8.9 ± 2.2	9.0 ± 2.1	0.350
LnHOMA-IR	0.9 ± 0.1	1.3 ± 0.1	0.022
25(OH)D (ng/ml)	18.6 ± 7.8	16.5 ± 6.8	0.001
TT (nmol/L)	9.3 ± 7.8	8.9 ± 7.3	0.482
E2 (pmol/L)	68.6 ± 3.9	72.5 ± 5.2	0.223

Data were expressed as mean ± *SD* or median (interquartile range). Non-normally distributed data were ln-transformed to normality before analysis. *P* < 0.05 were considered to be statistically significant. DM, diabetes mellitus; BMI, body mass index; SBP, systolic blood pressure; DBP, diastolic blood pressure; TC, total cholesterol; TG, triglyceride; HDL-C, high-density lipoprotein cholesterol; LDL-C, low-density lipoprotein cholesterol; FPG, fasting plasma glucose; FINS, fasting insulin; FCP, fasting C peptide; HOMA-IR homeostasis assessment model of insulin resistance; HbA1c, glycosylated haemoglobin; 25(OH)D, 25-hydroxyvitamin D; TT, total testosterone; E2, estradiol.

**Table 2:** Demographic and clinical characteristics of study cohort stratified by DR status and gender.

Parameters	Females (*n* = 194)			Males (*n* = 341)		
	NDR (*n* = 95)	DR (*n* = 99)	*P*-value	NDR (*n* = 165)	DR (*n* = 176)	*P*-value
Age (year)	62.6 ± 14.1	65.5 ± 8.1	0.049	58.1 ± 12.7	62.8 ± 9.1	< 0.001
DM duration (year)	11.4 ± 6.6	15.5 ± 8.1	< 0.001	9.5 ± 6.7	13.7 ± 7.3	< 0.001
BMI (kg/m^2^)	25.4 ± 4.5	24.4 ± 3.6	0.089	25.6 ± 4.6	24.9 ± 3.3	0.105
SBP (mmHg)	134 ± 20	139 ± 20	0.044	134 ± 22	138 ± 19	0.025
DBP (mmHg)	73 ± 9	71 ± 10	0.129	76 ± 12	75 ± 11	0.606
TC (mmol/L)	4.6 ± 1.1	4.7 ± 1.2	0.768	4.2 ± 1.1	4.1 ± 1.0	0.613
TG (mmol/L)	1.7 ± 1.2	1.8 ± 1.1	0.579	1.7 ± 1.0	2.1 ± 1.4	0.006
HDL-C (mmol/L)	1.3 ± 0.3	1.2 ± 0.4	0.498	1.0 ± 0.2	1.1 ± 0.3	0.103
LDL-C (mmol/L)	2.7 ± 1.1	2.8 ± 1.1	0.635	2.4 ± 0.8	2.4 ± 0.9	0.663
FPG (mmol/L)	8.1 ± 3.5	8.3 ± 3.1	0.770	8.4 ± 3.4	8.1 ± 3.1	0.359
FINS (mU/L)	14.7(15.0)	18.3(11.0)	0.003	13.6(8.9)	29.8(12.6)	0.012
FCP (mmol/L)	2.2(1.5)	1.9(1.4)	0.030	2.3(1.3)	2.1(1.4)	0.045
HbA1c (%)	8.8 ± 2.3	9.3 ± 1.9	0.198	8.9 ± 2.3	8.9 ± 2.1	0.919
LnHOMA-IR	1.0 ± 0.9	1.2 ± 1.0	0.023	0.9 ± 0.1	1.3 ± 0.1	0.007
25(OH)D (ng/ml)	17.2 ± 5.3	16.7 ± 5.2	0.212	21.0 ± 5.0	16.4 ± 5.6	0.001
TT (nmol/L)	0.9 ± 0.2	0.7 ± 0.2	0.633	14.2 ± 5.5	12.9 ± 5.2	0.035
E2 (pmol/L)	71.8 ± 8.7	65.3 ± 10.8	0.640	65.9 ± 3.1	79.8 ± 4.8	0.455

Data were expressed as mean ± SD or median (interquartile range). Non-normally distributed data were ln-transformed to normality before analysis. *P* < 0.05 were considered to be statistically significant. DR, patients with diabetic retinopathy; NDR, patients without diabetic retinopathy; BMI, body mass index; SBP, systolic blood pressure; DBP, diastolic blood pressure; TC, total cholesterol; TG, triglyceride; HDL-C, high-density lipoprotein cholesterol; LDL-C, low-density lipoprotein cholesterol; FPG, fasting plasma glucose; FINS, fasting insulin; FCP, fasting C peptide; HOMA-IR, homeostasis assessment model of insulin resistance; HbA1c, glycosylated hemoglobin; 25(OH)D, 25-hydroxyvitamin D; TT, total testosterone; E2, estradiol.

### Correlation of blood 25(OH)D level and diabetic retinopathy in patients with T2DM

**Figure 2:**
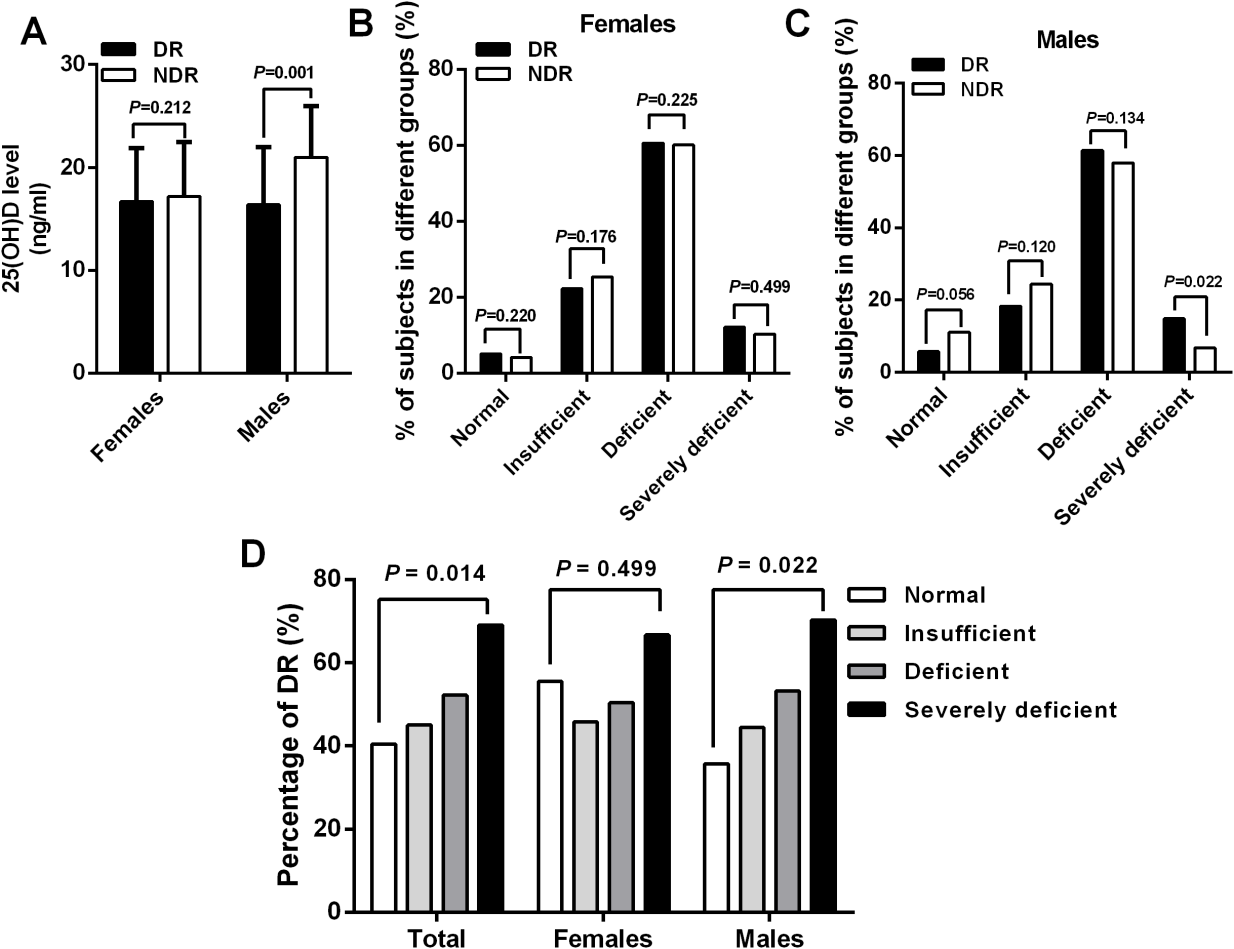
A significant decreased serum 25(OH)D levels **(A)** and increased percentage of severe 25(OH) D deficiency **(C)** were observed in male patients with DR than those without DR, which was not observed in females **(B)**. Similarly, there was a significantly increasing trend in the percentage of DR with the reduction of serum 25(OH)D levels in the total cohort (P = 0.014) and males (P = 0.022) but not in females (P = 0.499) (D).

In an attempt to explore the relationship between blood 25(OH)D concentration and risk of DR in patients with T2DM, we divided patients into four groups according to the serum 25(OH)D levels as follows: normal, insufficient, deficient, and severely deficient. Intriguingly, the results revealed that male participants with DR had significantly decreased levels of 25(OH)D (16.4 ± 5.6 ng/ml vs. 21.0 ± 5.0 ng/ml, *P* = 0.001) and increased proportions of severe 25(OH)D deficiency (14.8% vs. 6.7%, *P* = 0.022) than their counterparts, but such an association was not observed in females (**Figures 2A–C**). However, the prevalence of patients in normal, insufficient, and deficient groups did not differ significantly between DR and NDR groups in either gender (all *P* > 0.05). Similarly, there was a significantly increasing trend in the percentage of DR with the decrease of serum 25(OH)D levels in the total cohort and males (*P* = 0.014, *P* = 0.022, respectively), which was not observed in females (*P* = 0.499) (**Figure 2D**).

**Figure 3:**
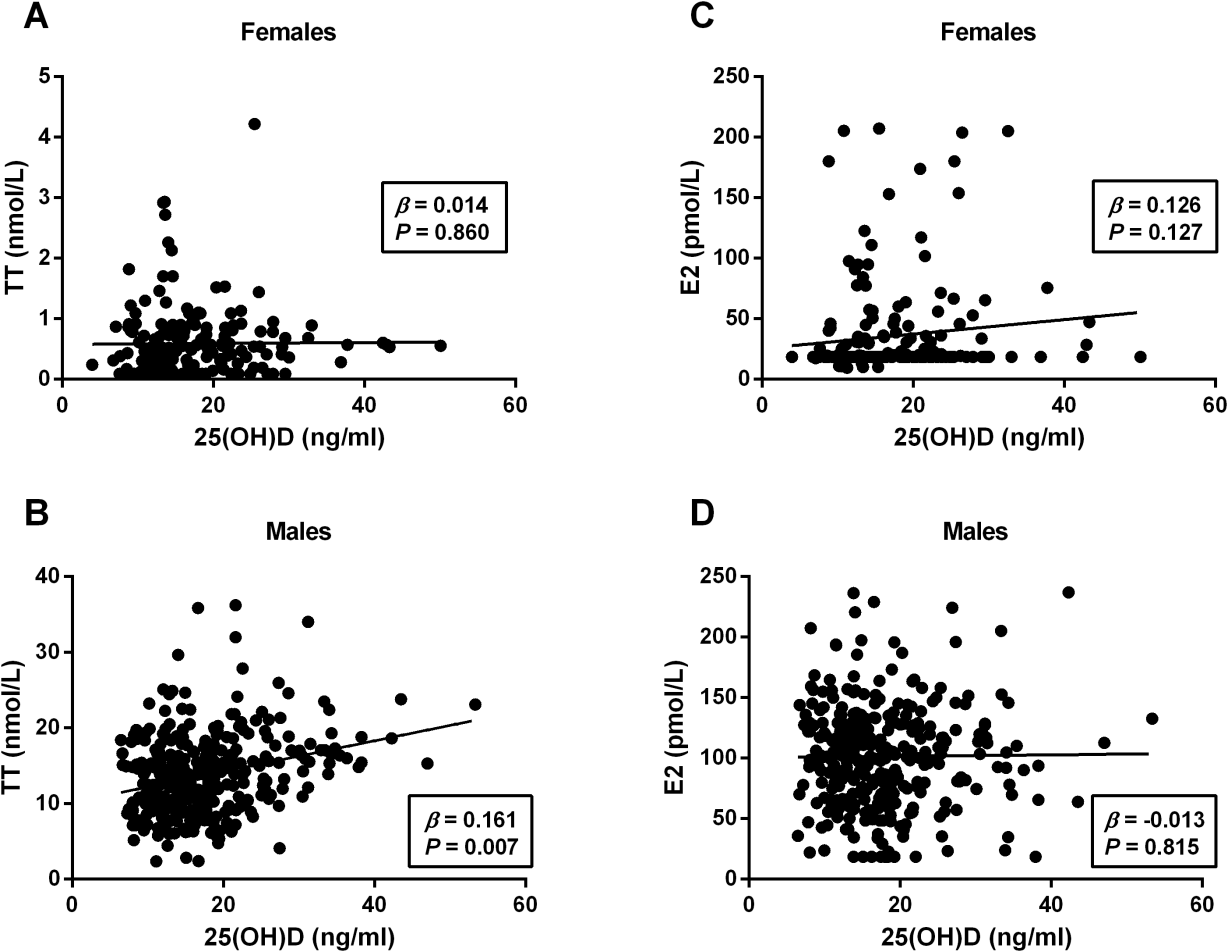
Linear regression analysis of serum 25(OH)D level with sex hormones in females and males. The 25(OH)D levels positively correlated with TT in males **(B)** but not in females **(A)**. No any significant correlation was observed between 25(OH)D and E2 in either gender (**C**, **D**). TT, total testosterone; E2, estradiol; 25(OH)D, 25-hydroxyvitamin **(D)** *P* < 0.05 were considered to be statistically significant.

To further evaluate the relationship between 25(OH)D levels and metabolic-related indices in patients with T2DM in both males and females, bivariate correlation analysis was performed (**Table 3**). Among females, 25(OH)D levels were found to be correlated negatively with BMI (*r* = −0.186, *P* = 0.010), TC (*r* = −0.150, *P* = 0.038), and TG (*r* = −0.148, *P* = 0.040) and positively with FCP (*r* = −0.153, *P* = 0.046). Among males, 25(OH)D levels correlated negatively with DM duration (*r*= −0.113, *P* = 0.038), TC (*r* = −0.120, *P* = 0.027), TG (*r* = −0.114, *P* = 0.036), and HOMA-IR (*r*= −0.156, *P* = 0.017), but positively with TT (*r* = 0.187, *P* = 0.001) and FCP (*r =* 0.172, *P* = 0.030). Moreover, multivariate linear regression analysis revealed that 25(OH)D levels were significantly associated with BMI (β = −0.192, *P* = 0.029) in females and TT (β = 0.161, *P* = 0.007), DM duration (β = −0.123, *P* = 0.039), TG (β = −0.142, *P* = 0.045), FCP (β = 0.163, *P* = 0.041), and HOMA-IR (β = −0.135, *P* = 0.033) in males but not correlated with age, DBP, SBP, TC, LDL-C, FPG, FINS, HbA1c, and E2 in either gender (all *P* > 0.05) (**Table 4**, **Figure 3**).

**Table 3:** Correlation of 25(OH)D levels with metabolic related indices in females and males.

Parameters	Females		Males	
	*R*	*P*	*r*	*P*
Age (year)	0.002	0.983	0.014	0.796
DM duration (year)	0.071	0.324	−0.113	0.038
BMI (kg/m^2^)	−0.186	0.010	−0.075	0.165
SBP (mmHg)	0.012	0.875	0.064	0.263
DBP (mmHg)	0.037	0.623	0.022	0.702
TC (mmol/L)	−0.150	0.038	−0.120	0.027
TG (mmol/L)	−0.148	0.040	−0.114	0.036
HDL-C (mmol/L)	0.027	0.706	−0.002	0.967
LDL-C (mmol/L)	−0.139	0.056	−0.105	0.054
FPG (mmol/L)	−0.121	0.094	−0.037	0.492
FINS (mU/L)	0.033	0.650	0.001	0.982
FCP (mmol/L)	0.153	0.046	0.172	0.030
HbA1c (%)	−0.053	0.469	−0.068	0.218
LnHOMA-IR	−0.116	0.185	−0.156	0.017
TT (nmol/L)	0.040	0.591	0.187	0.001
E2 (pmol/L)	0.117	0.113	0.011	0.834

BMI, body mass index; SBP, systolic blood pressure; DBP, diastolic blood pressure; TC, total cholesterol; TG, triglyceride; HDL-C, high-density lipoprotein cholesterol; LDL-C, low-density lipoprotein cholesterol; FPG, fasting plasma glucose; FINS, fasting insulin; FCP, fasting C peptide; HOMA-IR, homeostasis assessment model of insulin resistance; HbA1c, glycosylated hemoglobin; TT, total testosterone; E2, estradiol; 25(OH)D, 25-hydroxyvitamin D. *P* < 0.05 were considered as statistically significant.

**Table 4:** Multiple linear regression analysis of serum 25(OH)D with metabolic related indices in patients divided by gender.

Parameters	Females (*n* = 194)			Males (*n* = 341)		
	β	*t*	*P*	*B*	*T*	*P*
Age (year)	−0.019	−0.214	0.831	0.034	0.538	0.591
BMI (kg/m^2^)	−0.192	−2.203	0.029	−0.050	−0.773	0.440
SBP (mmHg)	−0.003	−0.042	0.967	0.087	1.147	0.252
DBP (mmHg)	0.038	0.467	0.641	−0.035	-0.458	0.647
TC (mmol/L)	−0.064	−0.263	0.793	−0.065	−0.610	0.542
TG (mmol/L)	−0.042	−0.434	0.665	−0.142	−1.337	0.045
LDL-C (mmol/L)	−0.085	−0.354	0.724	−0.006	−0.060	0.952
FPG (mmol/L)	−0.070	−0.609	0.543	0.041	0.569	0.570
FINS (mU/L)	0.018	0.173	0.863	0.009	0.131	0.896
FCP (mmol/L)	−0.079	−0.796	0.427	0.163	2.612	0.041
HbA1c (%)	−0.010	−0.098	0.922	−0.033	−0.481	0.631
LnHOMA-IR	−0.015	−0.131	0.896	−0.135	−1.148	0.033
DM duration (year)	0.051	0.559	0.577	−0.123	−2.075	0.039

BMI, body mass index; SBP, systolic blood pressure; DBP, diastolic blood pressure; TC, total cholesterol; TG, triglyceride; LDL-C, low-density lipoprotein cholesterol; FPG, fasting plasma glucose; FINS, fasting insulin; FCP, fasting C peptide; HbA1c, glycosylated hemoglobin; HOMA-IR, homeostasis assessment model of insulin resistance; DM, diabetes mellitus; 25(OH)D, 25-hydroxyvitamin D. *P* < 0.05 were considered as statistically significant.

### Influence of 25(OH)D on the occurrence of diabetic retinopathy in patients with T2DM

**Figure 4:**
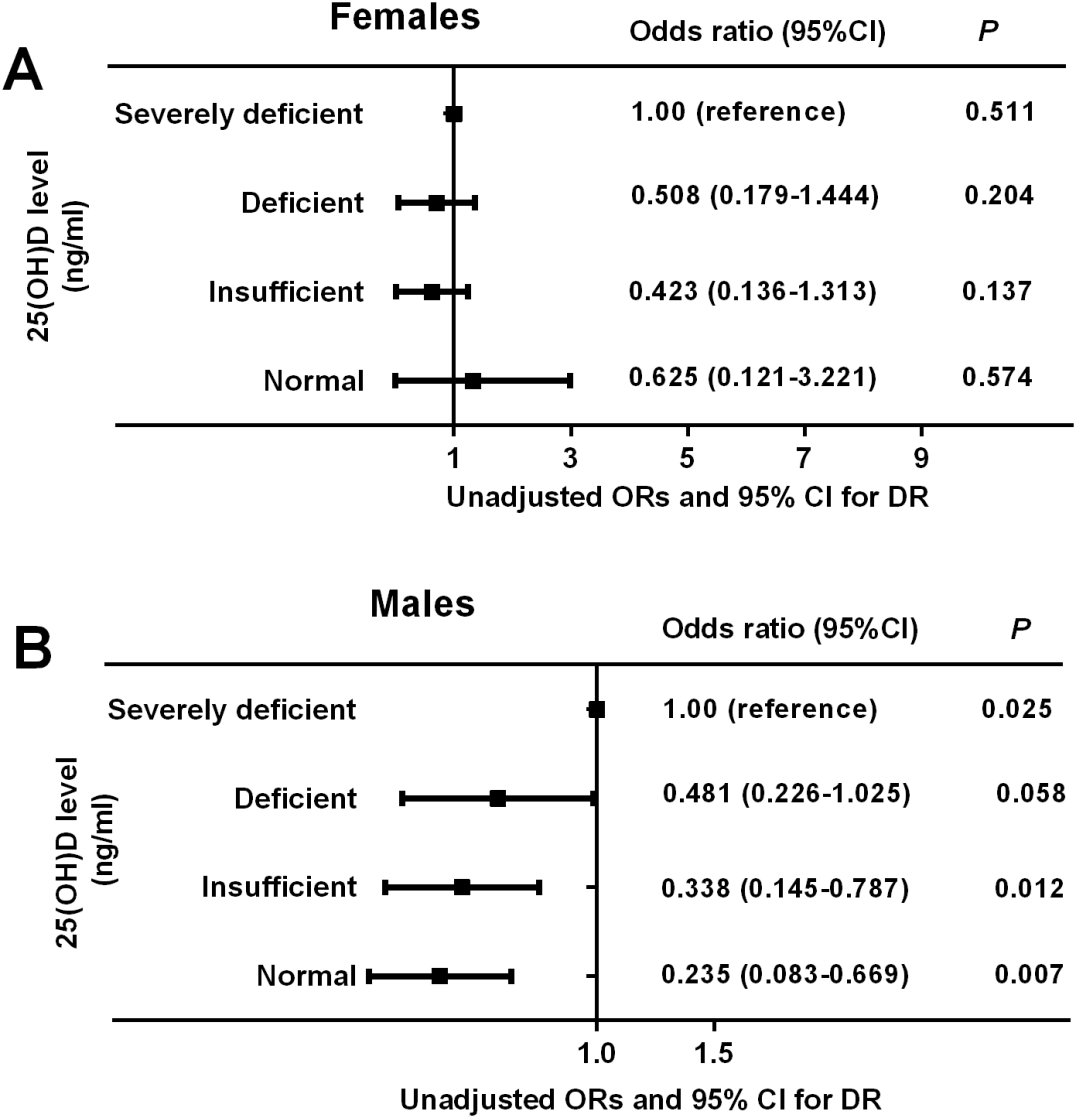
Unadjusted odds ratios (ORs) and 95% confidence intervals (CI) for DR based on serum 25(OH)D levels in females and males. The ORs (95% CI) for DR did not change significantly across the different 25(OH)D groups in females (*P*-trend = 0.214) **(A)** but reduced gradually across the different 25(OH)D groups in males (*P*-trend = 0.001) **(B)**. *P* < 0.05 were accepted as statistically significant.

To investigate the impact of serum 25(OH)D on the risk of DR in patients with T2DM, binary logistic regression analyses were used and the results demonstrated a significant gender difference between serum 25(OH)D and the risk of DR. Among males, we observed a significantly decreasing trend in the ORs and 95% CI for DR with the increment of 25 (OH)D levels, manifested by 0.481 (0.226–1.025, *P* = 0.058) in deficient group, 0.338 (0.145–0.787, *P* = 0.025) in insufficient group, and 0.235 (0.083–0.669, *P* = 0.025) in normal group when the severely deficient group was used as reference (*P_-trend_* = 0.001), whereas among females, the ORs (95% CI) for DR did not change significantly across the different 25(OH)D groups, manifested by 0.508 (0.179–1.444, *P* = 0.204) in deficient group, 0.423 (0.136–1.313, *P* = 0.137) in insufficient group, and 0.625 (0.121–3.221, *P* = 0.574) in normal group when comparing with the severely deficient group (*P_-trend_* = 0.214) (**Figure 4**). After adjusting for multiple potential confounders (age and BMI involved in Model 1; age, BMI, FPG, and FINS involved in Model 2; age, BMI, FPG, FINS, HOMA-IR, TT, and DM duration involved in Model 3), such an association remained significant in males but not in females (**Table 5**). The *P*-value for trend was significant in all regression models in males (0.001 in Model 1, 0.001 in Model 2, and 0.003 in Model 3), which was not detected in females (0.119 in Model 1, 0.117 in Model 2, and 0.137 in Model 3). Collectively, males but not females with T2DM showed a significant inverse association between serum 25(OH)D and the occurrence of DR, which might be mediated by the reduction in TT levels.

**Table 5:** Multiple-adjusted odds ratios (AOR) and 95% confidence intervals (CI) for DR based on serum. 25(OH)D levels: results of binary logistic regression analysis in different models.

25(OH)D groups	Model 1			Model 2			Model 3		
	β	AOR (95% CI)	*P*	β	AOR (95% CI)	*P*	β	AOR (95% CI)	*P*
Females									
Normal	−0.565	0.569 (0.108–2.992)	0.505	−0.564	0.569 (0.108–2.997)	0.506	−0.817	0.442 (0.079–2.474)	0.353
Insufficient	−0.931	0.394 (0.124–1.257)	0.116	−0.935	0.393 (0.123–1.258)	0.116	−1.149	0.317 (0.097–1.038)	0.058
Deficient	−0.677	0.508 (0.174–1.484)	0.216	−0.675	0.509 (0.174–1.487)	0.217	−1.043	0.353 (0.118–1.057)	0.063
Severely deficient		1.00 (reference)	0.473		1.00 (reference)	0.473		1.00 (reference)	0.260
*P* for trend			0.119			0.117			0.137
Males									
Normal	−1.497	0.224 (0.076–0.657)	0.006	−1.771	0.170 (0.053–0.543)	0.003	−1.457	0.233 (0.070–0.779)	0.018
Insufficient	−1.136	0.321 (0.135–0.765)	0.010	−1.451	0.234 (0.090–0.609)	0.003	−1.275	0.280 (0.103–0.756)	0.012
Deficient	−0.696	0.498 (0.229–1.085)	0.079	−0.928	0.395 (0.167–0.934)	0.034	−0.740	0.477 (0.196–1.164)	0.104
Severely deficient		1.00 (reference)	0.019		1.00 (reference)	0.006		1.00 (reference)	0.034
*P* for trend			0.001			0.001			0.003

AOR, adjusted odds ratios; 95% CI, 95% confidence intervals. Serum 25(OH)D levels were plotted into four groups as follows: Normal: ≥ 30 ng/ml; Insufficient: 20–30 ng/ml; Deficient: 10–20ng/ml; Severely deficient, < 10 ng/ml. Model 1: adjusted for age and BMI. Model 2: adjusted for age, BMI, FPG, and FINS. Model 3: adjusted for age, BMI, FPG, FINS, HOMA-IR, TT, and DM duration. *P* < 0.05 were considered to be statistically significant.

## Discussion

Our study revealed a marked inverse association between serum 25(OH)D and the risk of DR in male patients with T2DM but not in females, suggesting a significant gender-specific association between serum 25(OH)D and DR among the type 2 diabetic population. Such an association may actually be mediated by different changes in TT levels among DR patients, where TT levels were reduced significantly among males but not females. This work is, to the best of our knowledge, the first study to propose that sex hormones may differentially modulate vitamin D status and the risk of DR in men and women and provides a new insight into the correlation between serum 25(OH)D and DR incidence in diabetic patients, highlighting the need for gender-specific management strategies in T2DM with DR.

Epidemiological data has predicted a dramatic increment in the prevalence of DR, which is a devastating complication of T2DM (1), putting patients at high risk of visual impairment and blindness. It is strongly associated with multiple metabolic factors, including age at diagnosis, a longer DM duration, poor glycemic control, hypertension, insulin resistance (IR), hyperlipidemia, and cardiovascular diseases (2, 6, 27). Likewise, our study demonstrated that patients with DR tended to be elder and had significantly longer DM duration, higher SBP, TG, FINS, and HOMA-IR than their controls regardless of gender. Also, we observed a significantly decreased FCP level in patients with DR as opposed to those without DR. Consistently, the GOLDR (Genetics of Latino Diabetic Retinopathy) cohort study in Latinos with T2DM by Kuo et al. (28) and the cross-sectional study in Korean with T2DM by Kim et al. (29) confirmed an inverse association of DR with C-peptide concentration in T2DM. This indicates that endogenous insulin secretory impairment may be involved in the progression of DR (27). It might be explained by the fact that neural involvement is an important participant in the pathogenesis of DR (30), and insulin as a critical neuroprotective factor (31) may therefore protect against the development and progression of DR. Based on these metabolic disorders accompanied by DR, it is extremely crucial for us to determine key factors and potential mechanisms of DR among the diabetic population, aiming to provide clinical evidence for prevention and treatment.

Vitamin D, an essential nutrient element in bone metabolism, exerts a wide range of pleiotropic effects, including antioxidant defense (7), anti-angiogenesis (8), and immune and anti‐inflammatory functions (32), which are involved in the pathogenesis of DR (7). Totolici et al. (33) demonstrated a protective role of vitamin D on the development and progression of DR by reducing blood glucose, hypertension, and atherosclerosis. Likewise, evidence from randomized controlled trials revealed a significant reduction in fasting glucose levels, TG, and HOMA-IR, as well as an increase in HDL-C in vitamin D supplementation compared to placebo, suggesting that vitamin D may provide protection against the onset and progression of DR through its better glycemic control and amelioration of IR and dyslipidemia (15, 34). Nevertheless, available studies and meta-analyses have reported an inconsistent relationship between 25(OH)D deficiency and the occurrence of DR in diabetic patients. In the two large meta-analyses, individuals with DR had a significantly reduced 25(OH)D level and increased proportion of 25(OH)D deficiency compared to their controls, showing a significant inverse association between 25(OH)D and the risk of DR in T2DM (12, 13), which was in accord with the results from another prospective study by Chen et al. (11). By contrast, Reddy et al. (18) found that the proportion of 25(OH)D deficiency was significantly higher in diabetic patients with or without DR compared to those without T2DM, whereas no significant differences were observed between the groups with diabetes (66% and 63%, *P* > 0.05). Some other studies did not find any association between 25 (OH)D insufficiency and the presence or severity of DR in T2DM (19, 20). Noticeably, our study revealed a significant gender-specific association between serum 25(OH)D and the risk of DR among Chinese patients with T2DM. In male subjects, those with DR had significantly decreased levels of 25(OH)D and an increased proportion of severe 25(OH)D deficiency compared to those without DR, whereas such an association was not observed in females. Then, we stratified patients into four groups according to the serum 25(OH)D status and genders and showed that there was a significantly increasing trend in the percentage of DR with the reduction of 25(OH)D level in males but not in females. After adjusting for potential confounding factors (age, BMI, FPG, FINS, HOMA-IR, TT, and DM duration), the multivariate logistic regression analysis revealed a significant gender difference between serum 25(OH)D and the occurrence of DR. Among male participants, the AORs (95% CI) for DR gradually decreased with the increment of 25(OH)D levels, which was nonsignificant among females. These findings implied a dramatically contradiction with the previous research results and provided an interesting novel insight into the relationship between 25(OH)D and DR with significant gender difference. Similar gender-specific effects of vitamin D have been observed in autoimmune diseases such as systemic lupus erythematosus, where lower vitamin D levels are linked to more severe disease in females but not males (35). This suggests that sex hormones may differentially regulate vitamin D metabolism across diseases, warranting further research.

With regard to the role of 25(OH)D in the occurrence of DR, it has been studied in numerous studies, Al-Qahtani et al. (36) reported a significant association between vitamin D and C-peptide levels in T2D. In our study, we observed a significantly decreased 25(OH)D and FCP levels in patients with DR as opposed to their counterparts, indicating that 25(OH)D deficiency may be involved in the progression of DR through endogenous insulin secretory failure. It might be explained by the fact that neural involvement is an important participant in the pathogenesis of DR (30), and insulin as a critical neuroprotective factor (31) may therefore protect against the development and progression of DR (27). Other studies suggest that vitamin D protects against DR through its anti-angiogenic and anti-inflammatory properties. In the oxygen-induced ischemic retinopathy mice model, calcitriol-treated mice demonstrated a significantly inhibited retinal neovascularization in a dose-dependent manner compared to their controls, suggesting that calcitriol is a potent inhibitor of retinal neovascularization and may be of benefit in the treatment of DR (8). Proinflammatory cytokines such as IL-6 and TNF-α were found to be increased in patients with T2DM, and vitamin D supplementation could reduce the production of these proinflammatory cytokines in the retina by enhancing its anti-inflammatory properties (14). Moreover, vitamin D could prevent the expression of Toll-like receptors (37) and modulate genes encoding anti-inflammatory cytokines, therefore avoiding the inflammatory process. Furthermore, vitamin D can prevent oxidative stress-induced DNA damage supporting antioxidant defense (38), regulate mitochondrial dysfunction and the immune system, as well as cell activities such as angiogenesis, apoptosis, and autophagy (7), thus affecting the pathogenesis of DR. However, few data are available on the gender-specific relationship of 25(OH)D with DR incidence, thus warranting further investigation.

Due to that evidence concerning the gender difference of 25(OH)D level with the risk of DR among diabetic patients being lacking, the underlying mechanism remains unclear. Intriguingly, in the present study, we demonstrated that levels of TT were decreased significantly in male patients but not females with DR compared to their counterparts, whereas E2 levels did not change significantly between groups in either gender. To further identify the main factors that might influence the contributing role of serum 25(OH)D level on the risk of DR in diabetic patients, we performed the multivariate linear regression analysis. The results showed that 25(OH)D levels were positively associated with TT levels among male participants with T2DM, which was in line with the previous conclusion suggested by Brooke et al. (39). Also, relevant clinical and experimental evidence has revealed a significant positive association between 25(OH)D and TT (40, 41). In a large systematic review and meta-analysis including eighteen studies enrolling 9,892 men with vitamin D deficiency and 10,675 controls, D’Andrea et al. (41) showed a significant positive association between 25(OH)D and TT along with a noticeable decrease in heterogeneity, which could only be demonstrated in subjects with frailty states (95% CI: −0.27, −0.10, *P* < 0.0001; I2 = 51%, *P* for heterogeneity = 0.06). However, there was no significant association between 25(OH)D levels and E2 in either gender. In addition, 25(OH)D levels were found to be correlated positively with FCP and negatively with TG, HOMA-IR, and DM duration in males but negatively with BMI in females. Similarly, evidence from randomized controlled trials revealed a significant reduction in FBG, TG, and HOMA-IR, as well as an increase in HDL-C and FCP in vitamin D supplementation compared to placebo (15, 34, 42, 43). These findings thus indicate that testosterone deficiency might be a crucial contributing factor to hypovitaminosis D in the onset and progression of DR in addition to poor glycemic control, IR, dyslipidemia, and insulin secretion impairment, which was only observed in males. Unlike these studies, there are conflicting reports of their associations in others, which observed no significant association of 25(OH)D with TT in men (44, 45). Also, a recent meta-analysis of 10 randomized controlled trials did not find any evidence to support beneficial effect of vitamin D supplementation on TT levels in men (46). These discrepant results may be due to different diabetes status, ethnic variations, sample size, subgroup classification, or methodological differences among these studies. Notably, higher serum 25(OH)D levels were reported to be significantly associated with reduced risk of diabetic microvascular complications, including DR, diabetic nephropathy, and diabetic neuropathy, suggesting a potential beneficial role of maintaining adequate vitamin D status in the prevention of diabetic microvascular complications (11). However, our study did not include a comprehensive assessment of these comorbidities. Future studies should examine whether 25(OH)D deficiency is independently associated with DR or part of a broader metabolic disturbance in diabetes.

Although the exact underlying mechanisms by which low TT might mediate the relation between 25(OH)D deficiency and increased odds of DR in males with DR are unclear, several underlining reasons may account for this pronounced finding. To begin with, TT, as an important sex steroid hormone, has dramatically higher serum levels in men than women, which increases 30-fold by adulthood but remains low in females (47). In addition, low TT levels have been validated to be associated with multiple metabolic diseases with gender differences. Zhang et al. (48) found that low TT was independently associated with increased risk of NAFLD among men but not women with T2DM. Dong et al. (49) also found that low TT concentrations were associated with poor cognitive performance in older men but not women in the United States. Another prospective cohort study by Harris et al. (50) demonstrated that a higher estrogen:testosterone (O/T) ratio was associated with a lower risk of myocardial infarction after adjustment for CVD risk factors in men but not in women. Similarly, we found that TT levels were significantly reduced in male but not female patients with DR. Therefore, we hypothesize that low TT may play a crucial role in the development of DR in men but not women. Moreover, vitamin D, working as an actual steroid hormone, exerts pleiotropic biological effects not only in bone metabolism, which depends on the gene expression of the vitamin D receptor (VDR) (51). The VDR, almost ubiquitously expressed, is also expressed by human Leydig cells (52). Increasing evidence has highlighted a possible direct causal link between hypovitaminosis D and low testosterone. In the study by Hofer et al. (53), human Leydig cells were found to suffer from significant alterations in the expression profile of steroidogenesis genes after exposure to 1,25(OH)D3 *in vitro*, ultimately inducing a significant increase in TT synthesis in human testicular cell cultures. Furthermore, VDR-deficient mice were found to develop hypergonadotropic hypogonadism (54). In addition, the vitamin D metabolizing enzymes (CYP2R1, CYP27A1, and CYP27B1) are also widely expressed in Leydig cells of the testis, and vitamin D deficiency may result in reduced hypogonadism (55). Also, studies have reported that vitamin D pleiotropism may also be involved in the testicular functions, because of that human Leydig cells express the CYP2R1 gene, which encodes the expression of 25-hydroxylase, therefore converting cholecalciferol into 25(OH)D (56). In this scenario, we assume that 25(OH)D deficiency may contribute to the development of DR among male subjects with T2DM, directly or indirectly through the modulation of testosterone. While our study highlights the association between TT and 25(OH)D in males with DR, other sex hormones may also play a crucial role. Estrogen, for instance, has been shown to exert protective effects through improving retinal microcirculation and could interact with vitamin D metabolism (57). Inconsistently, Siddiqui et al. (58) found that estradiol, LH, and FSH levels were similar in participants with and without DR among pre- and post-menopausal women with T2DM and concluded that female sex hormone was not related to the presence of DR. Future investigations should explore the interplay between vitamin D and other sex hormones in DR pathogenesis.

Undeniably, there are some limitations to this study. One limitation is that our study featured a cross-sectional design, which might not reflect the causal relationship between serum 25(OH)D and DR in type 2 diabetic patients. It is possible that DR progression leads to lower vitamin D levels due to inflammation or reduced outdoor activity. Longitudinal studies and randomized controlled trials are needed to determine the causal direction of this association. Second, some other factors including seasonal variations, sunlight exposure, dietary vitamin D intake, and physical activity may influence the serum 25(OH)D levels, whereas these variables were not measured in our study, representing a limitation. Future studies should incorporate objective measures of these factors. Third, the measurement methods of circulating 25(OH)D levels and the diagnosis of vitamin D deficiency were incongruent among available studies, which may influence the study conclusion. Finally, another key limitation of our study is that it was conducted at a single center, and the sample size was relatively small, which may restrict the generalizability of our findings to broader populations. The vitamin D status and DR prevalence could vary based on geographical location, lifestyle, dietary patterns, and genetic background. Available data has validated that vitamin D level vary across countries/regions as well as even over time within a country. In the study by Sempos et at (59), they collated data from nationally representative surveys from individual countries and found a relatively low overall prevalence of vitamin D deficiency in South America (3%; 3%–21%), Oceania (5%; 5%–6%), and North America (7%; 5%–9%), whereas more moderate prevalence in Europe (13%) and Asia (18%; 3%–45%). These differences may be influenced by different sunlight availability, seasonal cycles, cultural practices, urbanization, and dietary policies (60). Therefore, future studies should include multi-center or multi-ethnic cohorts to validate our results and explore potential regional variations in the association between serum 25(OH)D levels and DR.

## Conclusion

This study concluded a gender-specific relationship between serum 25(OH)D and the risk of DR among patients with T2DM, as manifested by a marked inverse association between serum 25(OH)D and DR in males but not in females. Such an association may actually be mediated by different TT statuses among DR patients, where TT levels reduced significantly among males but not females. Although the roles of 25(OH)D and sex hormones in the development of DR are complex, these findings help us identify individuals at a higher risk of DR in T2DM who could benefit from individualized preventive and therapeutic strategies. Future studies should (1) confirm the causality between 25(OH)D deficiency and DR through longitudinal or interventional designs; (2) investigate the molecular mechanisms underlying vitamin D’s role in DR pathogenesis; (3) explore potential sex-specific interventions such as vitamin D and testosterone supplementation in male patients with DR; and (4) include multi-center, large-scale studies to validate these findings across different populations.

## Data Availability

The raw data supporting the conclusions of this article will be made available by the authors, without undue reservation.
